# Recent Advances of Tubulin Inhibitors Targeting the Colchicine Binding Site for Cancer Therapy

**DOI:** 10.3390/biom12121843

**Published:** 2022-12-10

**Authors:** Mohammed Hawash

**Affiliations:** Department of Pharmacy, Faculty of Medicine and Health Sciences, An-Najah National University, Nablus P.O. Box 7, Palestine; mohawash@najah.edu; Tel.: +970-569939939

**Keywords:** cancer, FDA, tubulin, discovery, polymerization, depolymerization

## Abstract

Cancer accounts for numerous deaths each year, and it is one of the most common causes of death worldwide, despite many breakthroughs in the discovery of novel anticancer candidates. Each new year the FDA approves the use of new drugs for cancer treatments. In the last years, the biological targets of anticancer agents have started to be clearer and one of these main targets is tubulin protein; this protein plays an essential role in cell division, as well as in intracellular transportation. The inhibition of microtubule formation by targeting tubulin protein induces cell death by apoptosis. In the last years, numerous novel structures were designed and synthesized to target tubulin, and this can be achieved by inhibiting the polymerization or depolymerization of the microtubules. In this review article, recent novel compounds that have antiproliferation activities against a panel of cancer cell lines that target tubulin are explored in detail. This review article emphasizes the recent developments of tubulin inhibitors, with insights into their antiproliferative and anti-tubulin activities. A full literature review shows that tubulin inhibitors are associated with properties in the inhibition of cancer cell line viability, inducing apoptosis, and good binding interaction with the colchicine binding site of tubulin. Furthermore, some drugs, such as cabazitaxel and fosbretabulin, have been approved by FDA in the last three years as tubulin inhibitors. The design and development of efficient tubulin inhibitors is progressively becoming a credible solution in treating many species of cancers.

## 1. Introduction

Cancer accounts for numerous deaths each year, and it is one of the most common causes of death worldwide, despite many breakthroughs in the discovery of novel anticancer candidates [1,2]. Each new year, the FDA approves the use of new drugs for cancer treatments, but due to multiple drug resistance and serious side effects, current treatments become non-ideal therapy; because of that, great efforts to discover a new agent with fewer toxic effects are necessary [3,4,5]. Many new chemical structures were designed and synthesized regarding cancer’s biological targets, such as cyclin-dependent kinase (CDK), epidermal growth factor (EGF), Ras, and tubulin proteins. These targets were classified as the main targets of new anticancer candidates [6,7], and with regards to this, tubulin is considered as one of the most useful and strategic molecular targets for antitumor drugs [8]. Microtubules play an important role in intracellular cell division, as well as in transportation. Tubulin protein polymerizes into long chains, or filaments, to build hollow fibers, or microtubules. These fibers work like a skeletal system for living cells and are the clear target for anticancer agents [9,10]. The targeting of tubulin protein by the inhibition of microtubule formation usually induces apoptosis (programmed cell death) [11,12].

The design and discovery of new tubulin inhibitors (TIs) targeting the colchicine binding site appears an attractive path for improving and advancing tubulin inhibitors [13]. TIs are less prone to develop multi-drug resistance (MDR) in comparison with vinca alkaloids and taxanes because they are poor substrates for efflux mechanism P-gp [14,15]. Moreover, many TIs have disadvantages, such as serious side effects like neurotoxicity, and chemical instability [16]. Currently, an FDA-approved drug, fosbretabulin (combretastatin A-4 phosphate), which is utilized for the treatment of thyroid cancer, can specifically target the colchicine binding site of tubulin [17,18].

Therefore, TIs that bind to the colchicine site has received extraordinary attention in the last 10 years [19]. Based on this data, numerous microtubule targeting agents have been discovered as effective TIs for various cancer forms in the last decade, and some of these chemicals have entered clinical trials. This review aimed to describe recent advances in the development of chemical structures that target tubulin at the colchicine binding site as promising anticancer agents.

## 2. Combretastatin A-4 Analogues

**Figure 1:**
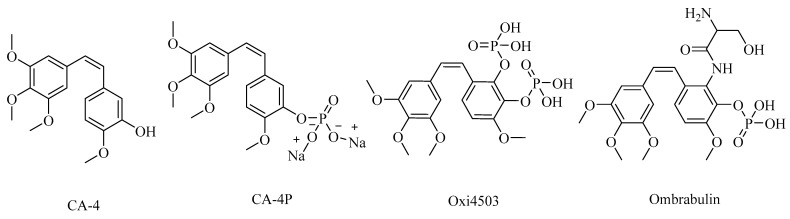
The structures of combretastatin A-4 (CA-4), fosbretabulin (CA-4P), Oxi4503 and Ombrabulin.

Combretastatin A-4 (CA-4) (Figure 1) is the most common member of the combretastatin family, which was isolated from the African tree Combretum caffrum [20,21,22]. **CA-4** exhibits strong antimitotic activity by binding to the colchicine binding site and entered phase II and phase III studies in clinical trials [23,24]. CA-4 has various pharmacokinetic disadvantages, such as poor water solubility [25,26,27], as well as having a short plasma half-life and instability due to isomerization from active cis isomer to inactive trans isomer under in vivo conditions [28,29]. To improve the low water solubility of CA-4, researchers developed CA-4, and optimized this pharmacokinetic challenge by innovating **CA-4P** (fosbretabulin) (Figure 1), which was applied for thyroid cancer, and was approved by the FDA in 2018 [17,30]. Moreover, many fosbretabulin salts were developed as fosbretabulin disodium and fosbretabulin tromethamine, as well as new derivatives being discovered, such as **Oxi4503** and **Ombrabulin** (Figure 1). This has been trialed as monotherapy, as well as in combination with well-known anticancer agents such as cisplatin, paclitaxel, carboplatin, pazopanib, and bevacizumab [31,32]. Recently, several studies have been performed on the CA-4 derivatives and their antiproliferative activities targeting tubulin were investigated [33,34,35,36,37].

**Figure 2:**
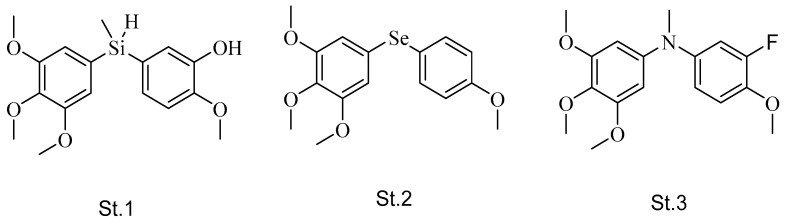
The structures of CA-4 analogues when the linker is replaced with hetero atoms (Si, Se, and N).

Researchers attempted to design and synthesize analogs of CA-4 by replacing the linker with hetero atoms (Si, Se, and N). Compound **st.1** (Figure 2) replaced the double bond between the two phenyl rings of CA-4 (linker) with a silicone atom; they were trying to design a compound that had a linker with a similar distance between two phenyl rings compared to CA-4. As a result, **st.1** showed inhibition of tubulin polymerization at 30 μM concentration, as well as the antiproliferative activities against breast cancer cell line (MCF7), with an IC_50_ value 7 nM [38]. In another study, the linker of CA-4 was replaced with selenide, and **st.2** (Figure 2) was active at nM concentration against MCF-7 cancer cell lines. This structure also showed potent activities as a tubulin polymerization inhibitor more active than CA-4 itself [39]. Soussi et al. synthesized CA-4 analogs methylated amine instead of CA-4 linker, and compound **st.3** (Figure 2) showed excellent anticancer activity at an average nanomolar level of mean GI_50_ values and inhibited tubulin assembly at a micromolar level. In addition, this compound, showed cell cycle arrest in the G2/M phase and induced apoptosis at very low concentration [40].

**Figure 3:**
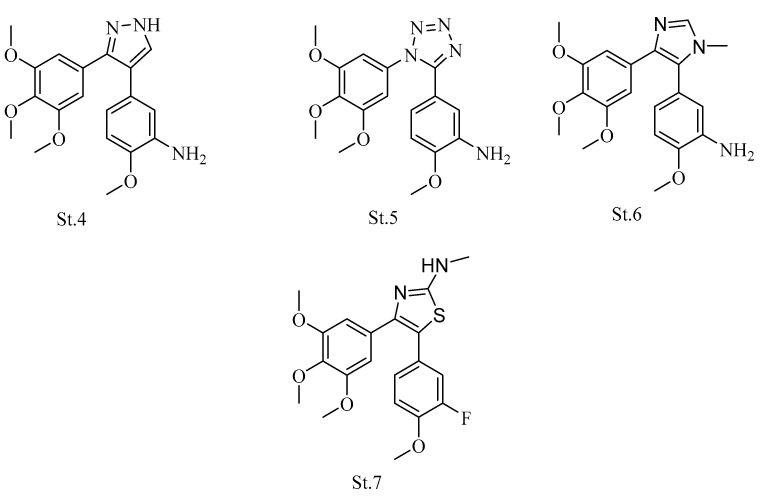
The structures of CA-4 analogues when the linker is replaced with heterocycles (pyrazole, tetrazole, and thiazole).

In various studies, researchers have placed heterocycle (pyrazole, isoxazole, tetrazole, thiazole, imidazole, pyrrole, oxazole, and β-lactam) instead of the double bound of CA-4 as a linker between phenyl rings. The incorporation of these heterocycles was very important for improving the water solubility of the CA-4 analogs [41,42,43,44,45,46]. Some introduced the pyrazole and tetrazole rings (**St.4** and **St.5**; Figure 3) instead of the double bond of CA-4; both of these structures showed tubulin polymerization inhibition with IC_50_ values 3 and 2 μM, respectively [34,47]. Wang et al. replaced the linker with an imidazole ring, compound **St.6** (Figure 3), with potent antiproliferative activities, and its pharmacokinetic properties were also perfect with 82% bioavailability in rats [48]. In another work, the thiazole ring was used as a linker, and it was substituted with methylamine. In terms of the structure-activity relationship, it was clear that NHCH_3_ substituent at the fifth position of the thiazole ring showed better antiproliferative activities than methyl or dimethylamine. However, in this series, compound **St.7** (Figure 3) was one of the most potent compounds against MCF-7 cancer cell lines with IC_50_ values in the nanomolar level, and it showed the inhibition of tubulin polymerization with IC_50_ 1.3 μM in comparison with CA-4 IC_50_ 1.2 μM [49].

**Figure 4:**
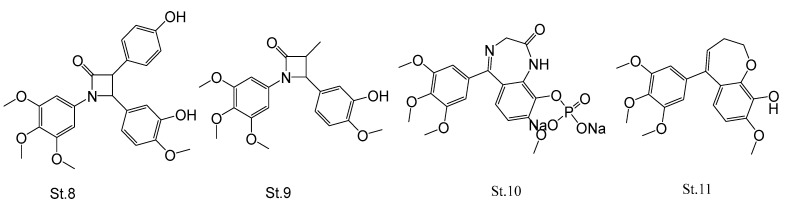
The structures of CA-4 analogues when the linker is replaced with heterocycles (four and seven-membered rings).

Several researchers have tried to use four-membered ring β-lactam as a bridge in the CA-4; these derivatives showed potent antiproliferative and antimitotic activities [50,51,52,53], and compound **St.8** (Figure 4) showed significant cytotoxicity against MCF7 cancer cell lines with IC_50_ values in nanomolar with significant in vitro inhibition of tubulin polymerization [54]. On the same core cycle, another group synthesized a series of (3-substituted 1,4-diaryl-2-azetidine) and compound **St.9** (Figure 4) was significantly the cell proliferation in IC_50_ range 31-63 nM, as well as its tubulin polymerization inhibition’s IC_50_ was around 3.5 µM. According to X-ray crystallography, this compound was binding to the colchicine binding site in tubulin in a similar mode like that of colchicine [55].

Seven membered rings as linkers were conducted in the synthesis of novel CA-4 derivatives, and one of these researches innovated a compound **St.10** (Figure 4), which inhibits the tubulin polymerization by binding to the colchicine binding site of tubulin. This compound showed potent antiproliferative activities against various kinds of human cancer cell lines, with IC_50_ values in nanomolar level, as well as the flow cytometric analysis results showing that this structure can induce cell cycle arrest in G2/M phase and apoptosis in A549 cancer cell line [56]. Another study was conducted on these basic core structures; a series of novel benzoxepins was synthesized and evaluated as anticancer agents, and among this series the most potent compound was **St.11** (Figure 4). This displayed antiproliferative activity with IC_50_ range 1.5-8 nM against different kinds of cancer cell lines (HCT116, K562, H1299, and MDA-MB231) and it also inhibited the tubulin polymerization at a micromolar range (IC_50_ = 3.8 µM) [57].

**Figure 5:**
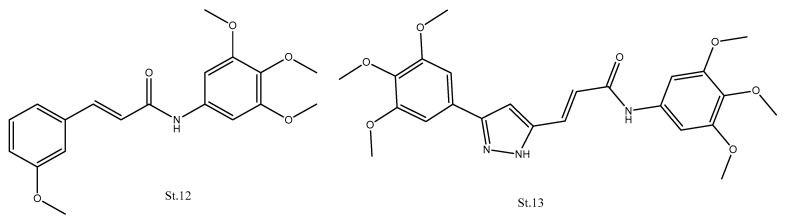
The structures of CA-4 analogues phenyl cinnamides and arylcinnamides.

Recent reports indicate that derivatives of pyrazole, isoxazole, and phenyl cinnamide, play an important role in the development of potential cytotoxic agents. In many kinds of research, the linker of CA-4 was replaced with amide or heterocyclic amide, and these derivatives showed significant antimitotic activities [58,59,60]. Phenyl cinnamides compound **St.12** (Figure 5) was shown to bind to tubulin, causing the inhibition of tubulin polymerization [61]. A series of pyrazole or isoxazole-linked arylcinnamides were designed and synthesized as anticancer agents; these compounds showed moderate to potent antiproliferative activities against HeLa, DU-145, A549, and MDA-MB231 cancer cell lines. In addition, among these structures, compound **St.13** (Figure 5) significantly depolymerizes tubulin with an IC_50_ value of about 1.5 μM. This compound was found to arrest the cells cycles in G2/M phase [62].

## 3. Indole Analogues

Many natural and synthetic compounds containing an indole ring showed various pharmacological activities, mainly anticancer properties, and many of these compounds target the tubulin protein to combat cancer cell proliferation [63,64,65].

**Figure 6:**
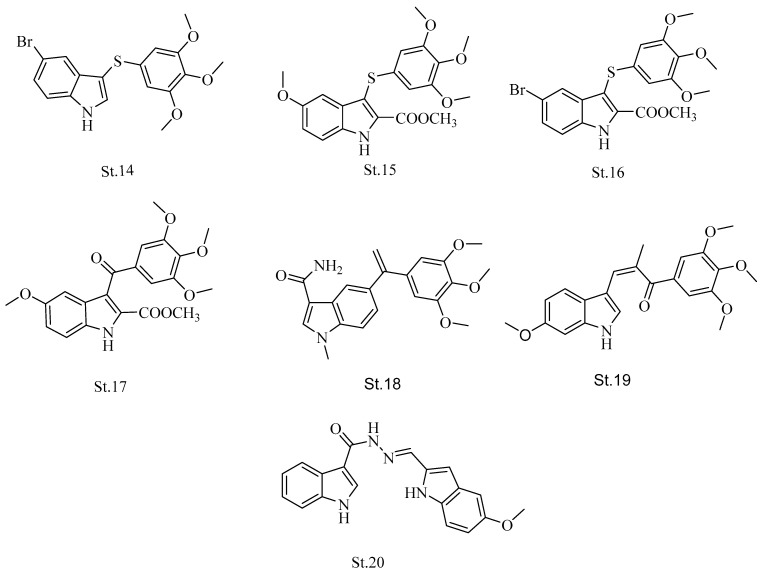
The structures of arylthioindole, trimethoxyphenyl-indole, and bis-indole.

The indole ring is considered a commonly distributed heterocycle in nature, and synthetic compounds, and it has been becoming an essential core structure in many pharmaceutical compounds. It possesses various pharmacological activities, such as antifungal, antioxidant [66], antiviral [67,68], antidepressant, anticonvulsant, and anti-inflammatory [65], and recently was involved in the discovery of new anticancer agents [69,70,71,72,73,74] as well as tubulin inhibitors [33,75,76]. Arylthioindole derivatives were one of the main classes of indole-containing compounds with tubulin inhibition activities. This family mainly contained trimethoxyphenyl moiety, beside the indole core structure [77]. **St.14, St.15, St.16,** and **St.17** (Figure 6) were potent tubulin inhibitors with IC_50_ values of 1.6, 2, 0.99, and 0.67 μM, respectively. They also exhibited antiproliferative activities with IC_50_ in nanomolar level against various cancer cell lines, such as MCF-7 and U937 [74]. Many structures were developed and discovered with various substitutes on the indole ring, and the trimethoxyphenyl moiety remained because of its supposed interactions with tubulin amino acids. One of these structures, **St.18** (Figure 6), exhibits potent antiproliferative activity with IC_50_ values in the nanomolar level; it also showed disruption of the microtubule network [78]. **St.19** (Figure 6) showed potent activity in various cancer cell lines without effects on normal cell lines [79]. Bis-indole derivatives were developed as antimitotic agents [80] and **St.20** (Figure 6) showed an inhibition effect on tubulin polymerization with an IC_50_ value of 7.5 µM, in addition to displaying anti-proliferative activity against the A549 cancer cell line with an IC_50_ value of 2 μM [81].

**Figure 7:**
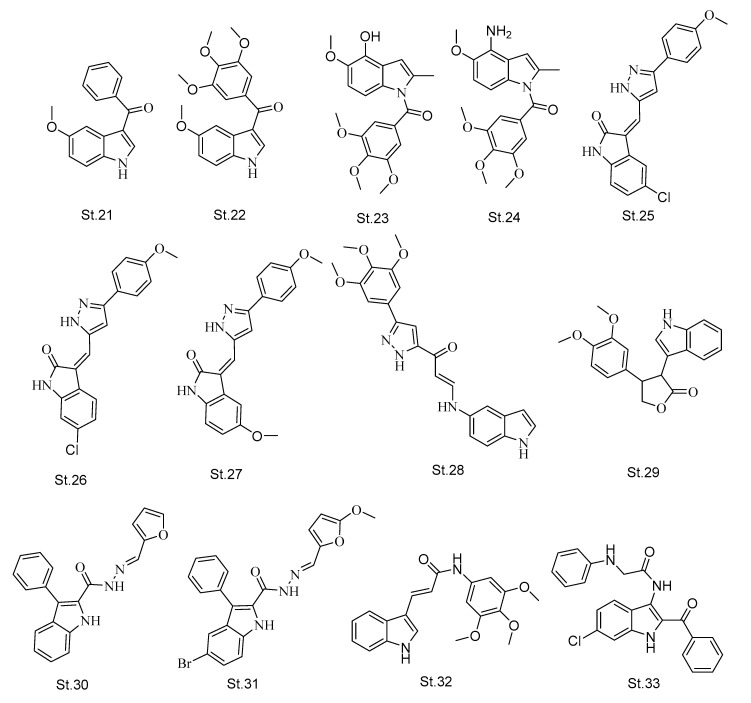
The structures of aroylindoles, trimethoxyphenyl-indole, indolyl-phenylmethanone, pyrazole-oxyindole, indole-amino-pyrazolyl, and indole-heterocyclic hybrid.

Aroylindoles have also been considered as one of the major classes of indole-containing compounds with tubulin-inhibiting effects [82]. **St.21** (Figure 7) was developed as a tubulin inhibitor agent and it interfered with the colchicine binding site of tubulin [83]. Additionally, **St.22** (Figure 7) is an indolyl-phenylmethanone derivative and showed potent antimitotic effects in human cancer cell lines, as well as potent antiproliferative activities against various kinds of cancer cell lines such as glioblastoma, breast, and gastric cancer cells [84]. In another study, aroylindole derivatives were discovered and synthesized as potent antitumor and antimitotic agents, **St.23** and **St.24** (Figure 7), with OH and NH2 at the 4th position of indole showing potent anti-tubulin activity with IC_50_ values of 0.6 and 0.9 μM, respectively. They also showed antiproliferative activity with IC_50_ in nanomolar level against different kinds of cancer cell lines [64,85].

Many kinds of research have been focused to discover hybrid chemical agents with antimitotic activities [86], including indole-heterocycles hybrids derivatives with promising anticancer activities [87,88]. Pyrazole-oxindole derivatives were also developed and evaluated on tubulin polymerization, and different kinds of cancer cell lines such as HeLa, A549, and MCF7, **St.25, St.26,** and **St.27** (Figure 7), exhibited anti-tubulin activities with IC_50_ values in the range 5.90–9.20 μM [89]. Meanwhile, **St.28** (Figure 7) with indole-amino-pyrazolyl derivatives was synthesized and inhibited tubulin polymerization with IC_50_ values of 0.28 μM [90]. In new research on a indole-furanone hybrid, derivatives were synthesized and developed as anti-tubulin derivatives; the most potent compound, **St.29** (Figure 7), has anticancer and antimitotic potency at the micromolar level [91]. In another recent work, the authors developed and synthesized an indole-heterocycle hybrid like the furan ring, and these structures showed potent antiproliferative activities. Among the synthesized compounds, **St.30** (Figure 7) was able to induce cell cycle arrest at the G2/M phase on A549 cancer cell line. Additionally, this structure also exhibited tubulin polymerization inhibitory activities, whereas when the bromine substitution was added to indole ring and methoxy on furan ring (**St.31**; Figure 7) and the anticancer activities were better than **St.30** with IC_50_ value under 0.5 μM against HuCCA-1 and HepG2 cancer cell lines [92].

The indole-amide class was considered one of the main classes of indole derivatives with anticancer and/or anti-tubulin activities [22,91,93]. A series of indole-acrylamide was synthesized and developed as anti-tubulin inhibitors, and **St.32** (Figure 7) showed inhibitory activities of tubulin polymerization. As well as this compound causing cell cycle arrest at the G2/M phase of HeLa and HL-60 cancer cell lines, it also induced apoptosis by the activation of caspase-3 [22]. The same group of researchers tried to target the tubulin by adding various substituents, such as methyl, nitrile, carboxylic acid, and ester, to the linker of **St.32.** However, the derivative with the nitrile group was considered as a tubulin polymerization inhibitor [94]. Another novel series of indole-based oxalamide was developed and designed, amongst which **St.33** (Figure 7) exhibits potent anticancer activities against HeLa, PC-3, and HCT-116 cancer cell lines. Meanwhile, the immunocytochemistry observed a significant loss of microtubule contents after the treatment of the cells with the compound, and confirmed the inhibition of tubulin polymerization accordingly [95].

## 4. Thiophene and Quinolone Analogs

**Figure 8:**
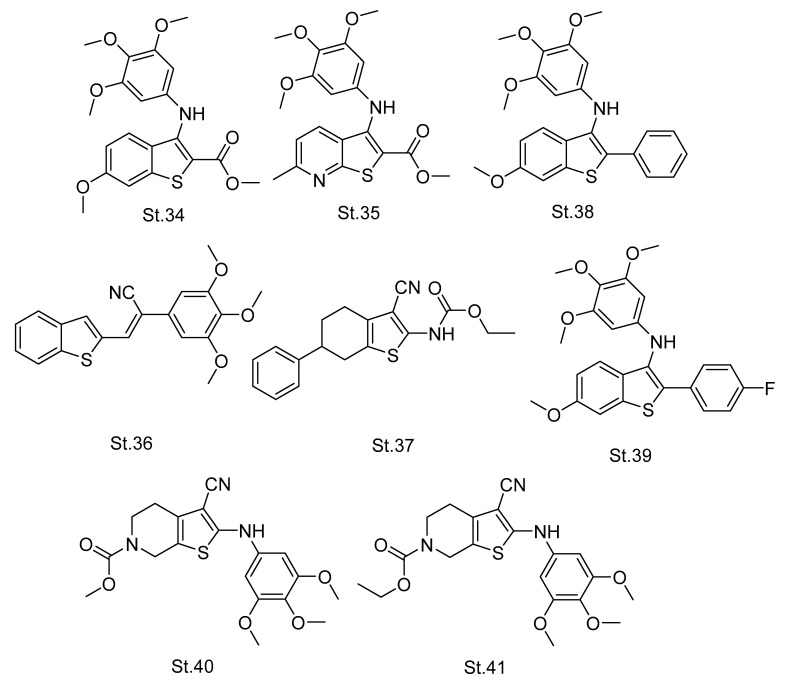
The structures of thiophene analogues.

Compounds containing thiophene rings have different biological activities, such as anticancer and anti-tubulin. A great deal of work has reported that the thiophene nucleus is an important structural heterocycle in antimitotic compounds [96,97,98]. A series of thiophene derivatives were designed, synthesized, and evaluated for anticancer activity against various cancer cell lines, as well as showing the inhibition of tubulin polymerization. Among the synthesized series both compounds **St.34** and **St.35,** (Figure 8), showed IC_50_ values of less than 1 nM against HeLa, HL-40, MCF-7, and HT-29 cancer cell lines, while the IC_50_ of tubulin polymerization was 0.88 and 0.70 μM, respectively [99]. In another study, 15 novel compounds were synthesized and evaluated against 60 kinds of cancer and normal cell lines, and the most potent compound was **St.36** (Figure 8), with benzothiophene moiety; IG_50_ values of this compound were less than 10 nM against most of tested cancer cell lines, and the IC_50_ value towards the inhibition of tubulin polymerization was 1.7 µM [100].

In a recent study, a series of tetrahydrobenzo[b]thiophene derivatives were synthesized and evaluated against colorectal cancer. The most active compound, **St.37** (Figure 8), showed moderate antiproliferative activities with IC_50_ values of 81.50 and 71.00 μg/mL against LoVo cells and HCT-116 cells cell lines. Meanwhile, molecular docking analysis in the colchicine binding site supposed good binding affinity of this compound [101]. Another new novel series of thiophene derivatives were synthesized and evaluated as anticancer agents and some of these compounds were considered significant apoptosis-inducing compounds. Compounds **St.38** and **St.39** (Figure 8) showed the greatest anticancer activity against HeLa and HT-29 cancer cell lines, with IC_50_ values of 0.06–0.50 μM. The colchicine binding studies were conducted to evaluate the tubulin polymerization inhibition, and the most active compound among the synthesized series was **St.39,** with a moderate inhibition percentage (30%) at 5 μM concentration in comparison with CA-4, with a potent inhibition percentage (97%) of the binding to [3H]colchicine to tubulin [102]. Another series of tetrahydrothiophene were designed, synthesized, and evaluated for anticancer activities against cancer cell lines and the inhibition of tubulin polymerization, as well as the effect on cell cycle phases. Compounds **St.40** and **St.41,** (Figure 8), with trimethoxyanilino and nitrile groups as important groups for the activities, these two compounds exhibited significant antiproliferative activities against L1210, CEM, and HeLa cancer cell lines with IC_50_ values in range 1.10–4.70 µM. They showed cell cycle arrest and accumulation at the G2/M phase and their IC_50_ values regarding the inhibition of tubulin polymerization were 3.8 and 3.4 µM, respectively, compared to the CA-4 IC_50_ value of 0.54 µM [103].

Quinoline heterocycle considers one of the important heterocycles, which possess various biological activities including anticancer, antimicrobial, anti-HIV, and anti-inflammatory [104]. In the last years, new chemical series containing quinoline and quinoline isosteres were designed to have antiproliferative activities [105,106] and target microtubules [107,108,109].

**Figure 9:**
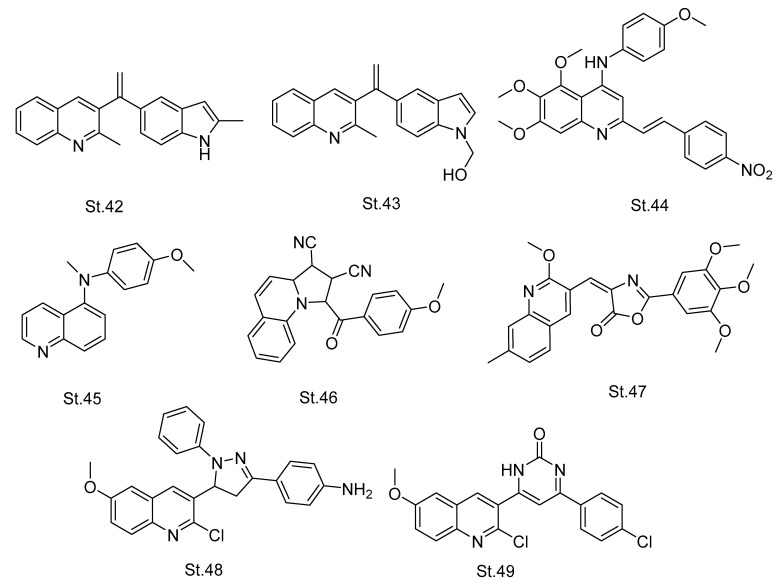
The structures of quinolone analogues.

A series of quinoline-indole derivatives were designed and synthesized to target tubulin and inhibit its functions, this series was a CA-4 analog. Additionally, the main core structure is similar to **St.18** (Figure 6); two compounds, **St.42** and **St.43** (Figure 9), showed the most potent antiproliferative against various cancer cell lines (HepG2, KB, HCT-8, MDA-MB-231, and H22), with IC_50_ values < 10 nM. Both of these compounds (St.42 and St.43) also effectively inhibited the tubulin polymerization with IC_50_ values 2.54 and 2.09 μM, respectively, in comparison with CA-4 IC_50_ 2.12 μM [108]. In recent work, researchers innovated a quinoline series by using the three-dimensional quantitative SAR strategy, and these series were virtually designed and evaluated as new anticancer/tubulin inhibitor agents. **St.44** (Figure 9) was one of the most active ligands regarding its possible binding interactions with the colchicine binding site [110].

Another series of quinoline derivatives were developed, synthesized, and evaluated as anticancer/tubulin inhibitors, amongst the synthesized series. **St.45** (Figure 9) was the most potent compound against HepG-2, B16-F1, HeLa, and MCF-7 cancer cell lines with IC_50_ value ranging from 0.261–2.047 μM. Furthermore, this compound has significant inhibition towards tubulin polymerization with IC_50_ 12.38 μM in comparison with CA-4 IC_50_ 1.84 μM [111]. In new work, cyano-pyrrolo-quinoline derivatives were synthesized and evaluated for their antiproliferative activity against 60 human cancer cell lines, and the most active compound, **St.46** (Figure 9), has GI_50_ values > 2.0 μM against most of the tested cancer cell lines. Additionally, in vitro assays and molecular docking studies regarding this compound found a significant binding interaction with tubulin [112]. In a recently published work, a new novel series of quinoline CA-4-based analogs were designed and evaluated as anticancer agents. One of the synthesized compounds, **St.47** (Figure 9), was considered a tubulin polymerization inhibitor by the performed mechanistic studies, and regarding cell cycle analysis it was made in accumulation and arrest in G2/M phase. In addition, this compound was the most active compound against MCF-7, HL-60, HCT-116, and HeLa cancer cell lines with IC_50_ values < 42 nM [113]. In a series of quinoline-pyrazole and quinoline-pyridone derivatives, **St.48** and **St.49** (Figure 9) showed the most potent tubulin polymerization inhibitory activities with IC_50_ values of 9.11 and 10.5 nM, respectively. These two structures showed significant antiproliferative activities against MCF-7, HepG-2, and HCT-116 cancer cell lines [114].

## 5. Chalcone Analogs

**Figure 10:**
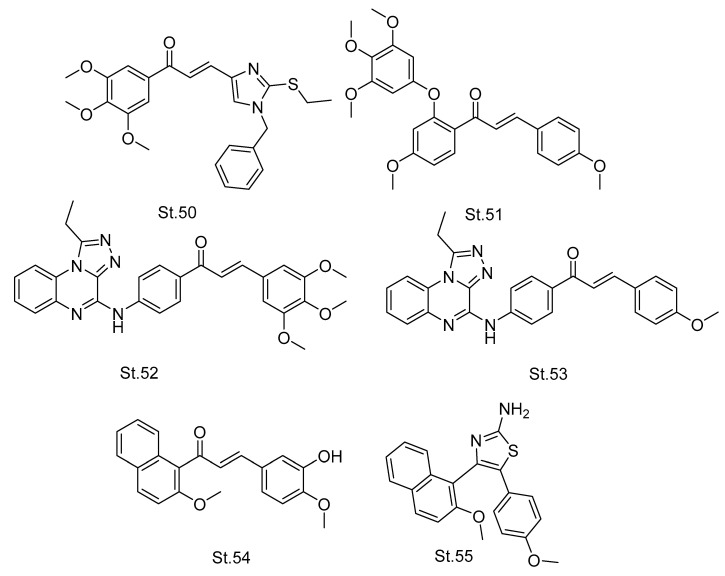
The structures of chalcone derivatives.

Chalcones (1,3-diaryl-2-proper-1-ones) are naturally occurring precursors of flavonoids, and these compounds have broad pharmacological activities including anti-cancer, antifungal, anti-inflammatory, and antioxidative activity [97,115,116,117,118]. A novel series of imidazole-chalcone derivatives were developed, synthesized, and evaluated as tubulin inhibitors. The most potent anticancer agent amongst the developed derivatives was **St.50** (Figure 10), A549, and MCF-7 cancer cell line with IC_50_ values of 7.05 and 9.88 μM, respectively. However, this compound also inhibited tubulin polymerization in a similar mode to CA-4 [119]. In another study, diaryl chalcone derivates were synthesized, and compound **St.51** (Figure 10) was the most potent anticancer agent against HCT116, HepG2, and MCF-7 cancer cell lines, with IC_50_ values < 6.31 μM. Additionally, the tubulin polymerization assay and molecular docking analysis supposed that this compound could effectively inhibit tubulin polymerization, and bind very well in colchicine binding site [120]. In another work, triazolo-quinoxaline chalcone derivatives were developed and two compounds, **St.52** and **St.53** (Figure 10), exhibited potent antiproliferative activities against MCF-7, HCT-116 and HepG2 cancer cell lines with IC_50_ range 0.84-15.4 μM. They could inhibit the EGFR with IC_50_ values of 39 and 83 nM, respectively; additionally they inhibit the tubulin polymerization with IC_50_ values 8.84 and 14.7 μM [109].

In another study, a series of naphthalene-chalcone derivatives were developed, synthesized, and evaluated as anticancer agents. Almost all of the synthesized derivatives showed considerable anticancer activities against the MCF-7 cancer cell line, and the most potent compound was **St.54** (Figure 10), with an IC_50_ value of 1.42 µM, with lower cytotoxicity against normal cell line Hek293t. Additionally, this compound inhibits the tubulin polymerization with IC_50_ value around 8 µM, in comparison with colchicine IC_50_ value (10.6 µM) [121]. By developing **St.54,** a series of naphthalene-thiazole derivatives were designed and among the synthesized compounds, **St.55** (Figure 10) was the most potent compound against MCF-7 and A549 cancer cell lines with IC_50_ values > 1 μM. It was found significant to inhibit the tubulin polymerization with IC_50_ value 3.3 µM [122].

## 6. Trimethoxy Phenyl Analogs

**Figure 11:**
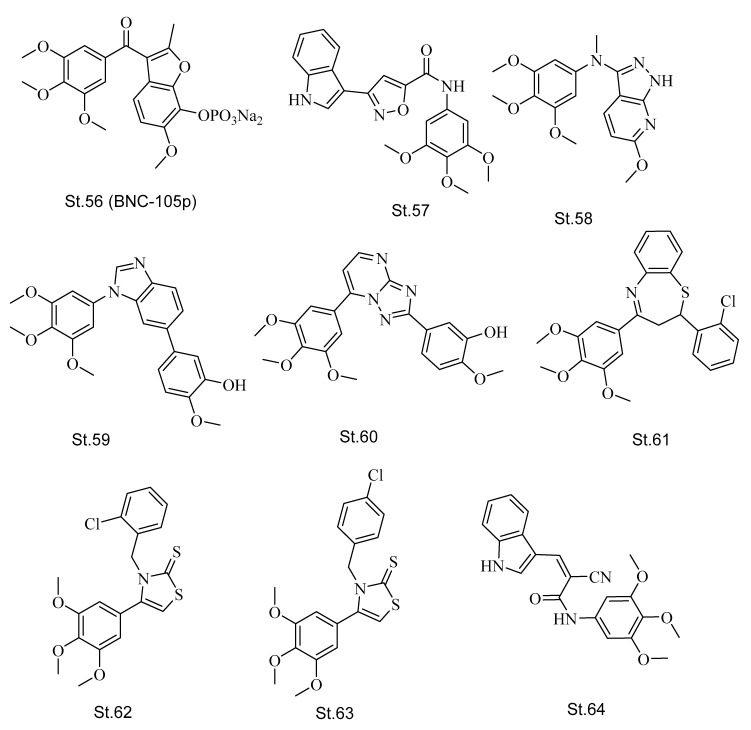
The structures of trimethoxyphenyl containing derivatives.

In the last decade, many trimethoxyphenyl-based structures were designed, developed, and synthesized as promising anticancer agents that could target tubulin protein, and some of these compounds reached clinical trials, or were approved by the FDA for cancer treatment [123,124,125]. Starting with CA-4P (Figure 1), which was approved for thyroid cancer, many other trimethoxyphenyl-based compounds entered the clinical trials for specific cancer types including **St.56** (BNC-105p; Figure 11), which was found to have considerable potency and an inhibitory effect against the growth of different kinds of cancer cell lines with a broader therapeutic index than CA-4P in vivo. It entered phase I of metastatic renal cell carcinoma malignant [126].

However, a lot of work with structures containing trimethoxyphenyl moiety were mentioned in the previous sections, and various structures with this moiety were designed and synthesized as promising anticancer agents. A series of isoxazole-carboxamide derivatives, such as **St.57** (Figure 11), exhibited potent antiproliferation activities against a panel of cancer cell lines; potent anticancer activities of St.57 was observed against Huh7, MCF7, and HCT116, with IC_50_ values 0.7, 3.6, and 1.3 μM, respectively, as well as potent activities against another hepatocellular carcinoma cell lines such as HepG2, Mahlavu, and SNU475 cancer (IC_50_ < 3.1 μM) [127]. Recently, many works focused on this moiety, and in a series of trimethoxyphenyl-pyrazolo-amine, **St.58** (Figure 11) was the most potent compound. The free amine group was supposed to play an essential role in the antiproliferative effects, and this compound exhibited significant anticancer activities against MCF-7, HCT-116, and HeLa cancer cell lines with IC_50_ values < 0.26 μM. Additionally, the mechanistic studies showed considerable inhibition towards tubulin polymerization activity with IC_50_ value of 14 μM [128]. In another recent study, the trimethoxyphenyl moiety with benzimidazole was used as CA-4 based structure, and compound **St.59** (Figure 11) exhibited the most potent effects against MCF-7, SGC-7901, and A549 cancer cell lines with IC_50_ values < 0.20 μM. At the same time, this compound inhibited tubulin polymerization by disrupting the cell microtubule networks, and cell cycle arrests were observed in G2/M phase. Regarding the in vivo study, this compound exhibited potent antitumor efficacy [129]. In a similar study to the previous one, researchers changed the benzimidazole of **St.59** with triazolo-pyrimidine, and in this series **St.60** (Figure 11) showed potent antiproliferative activities against a panel of cancer cell lines and specifically against HeLa cancer cell line with IC_50_ value of 0.06 μM and cell cycle arrest was observed in the G2/M phase. However, this compound inhibited tubulin polymerization with IC_50_ 1.3 μM, which was better than the positive control CA-4 IC_50_ value (4.22 μM) [130].

In another study with trimethoxyphenyl moiety, a series of the seven-membered ring (benzothiazepine), which is similar to CA-4 analogs (**St.10** and **St.11**; Figure 4), and compound **St.61** (Figure 11) was the most potent compound among this series with significant antiproliferative activities against MCF-7, HeLa, Ht29, and A549 cancer cell lines. The IC_50_ values were < 2 μM, as well as this compound inhibiting tubulin polymerization with IC_50_ values of 1.20 μM [131]. A series of thiazole-thiones containing trimethoxyphenyl moiety was designed and developed recently as anticancer agents. Among the synthesized derivatives, compounds **St.62** and **St.63** (Figure 11) were the most potent structures against the MCF-7 cancer cell line with IC_50_ values of 1.14 and 2.41 μg/mL, respectively. Regarding the obtained results of tubulin polymerization inhibition, St.62 was more potent than **St.63** with IC_50_ values 5.14 and 9.97 μg/mL, respectively [132]. In the newest work, a series of indole-acrylamide were developed and synthesized as promising antimitotic agents, with the antiproliferation activities of the synthesized compounds against a panel of cancer cell lines, and particularly focusing on hepatocellular carcinoma. Among this series, compound **St.64** (Figure 11) exhibited potent antiproliferative activities. Additionally, this compound, was determined to be a tubulin polymerization inhibitor with IC_50_ 18 μM. Furthermore, cell cycle arrest was observed in the G2/M phase in the Huh7 cancer cell line [94].

## 7. Approved and Promising Antimitotic Agents

In 1963, the first tubulin targeting drug (vincristine) was approved by the FDA for the treatment of cancer. Researchers began to take an interest in this class and many structures and drugs were discovered and approved for this purpose [133]. However, in the last decades, many TI agents have entered clinical trials and shown promising anticancer activities and some of these agents were approved to be used for certain kinds of serious cancers. Table 1 shows a list of drugs in clinical developments beside approved drugs that targeted tubulin as an anticancer agent [134].

## 8. Conclusions

In the last decades, great efforts have been made to discover tubulin inhibitors, and a few drugs have been approved by the FDA for the treatment of cancer by targeting tubulin as a molecular target. Unfortunately, most of the approved drugs for this target were associated with disadvantages, including low potency, drug resistance, and/or toxicity. However, because of these reasons, researchers are continuously attempting to develop and discover agents with ideal properties. Several groups were reached in this review article, and it was clear that the most important groups were CA-4 analogs, trimethoxyphenyl, and indole derivatives, and they exhibited potent antiproliferation and anti-tubulin activities. Various compounds with anti-tubulin activities were synthesized and developed, and it was clear that the compounds, which are similar to the CA-4 core structure and contain trimethoxyphenyl and indole, have very significant and considerable activities. In summary, this study focused on recent tubulin inhibitors, and the development of compounds with better selectivity, potency, and pharmacokinetic characteristics, will perhaps continue to receive fundamental attention in the following years, the results of which will change the perception of cancer treatment.

## Figures and Tables

**Table 1:** List of tubulin inhibitors in clinical developments or approved by FDA.

Drug Name	Chemical Structure	Type of Cancer	Microtubule	Status
Paclitaxel [135]	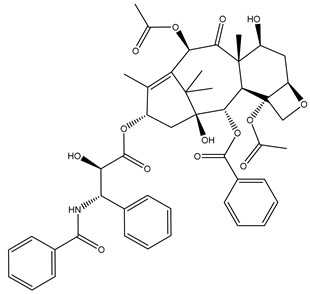	Metastatic adenocarcinoma of the pancreas	Stabilizing	Approved in 1998
Ixabepilone [136]	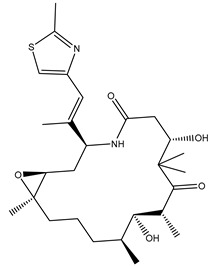	Metastatic or locally advanced breast cancer	Microtubule-stabilizing	Approved in 2007
Eribulin [137]	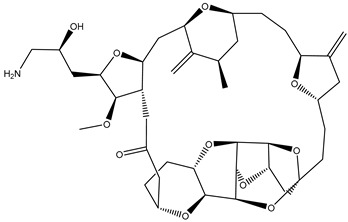	Recurrent metastatic breast cancer	Microtubule-destabilizing	Approved in 2010
BNC105P [19]	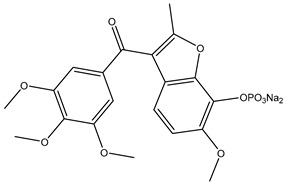	Leukemia	Inhibit polymerization	Phase I clinical trials
Plinabulin [138]	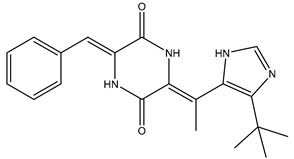	Lung Cancer	Inhibit polymerization	Phase I clinical trials
Tesetaxel [139]	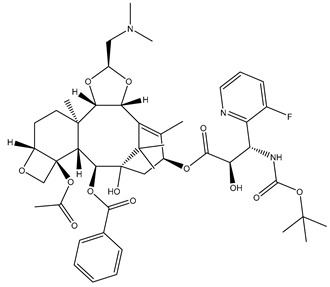	Breast Cancer	Stabilizing	Phase III clinical trials
KX2-361 [140]	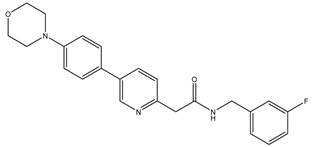	Solid Tumor	Inhibits polymerization	Phase I clinical trials
Fosbretabulin [18]	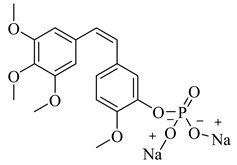	Thyroid cancer	Microtubule-destabilizing	Approved in 2018
Cabazitaxel [141]	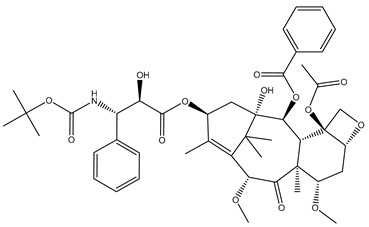	Metastatic, hormone-resistant prostate cancer	Stabilizing	Approved in 2019
Monomethyl Auristatin E [142]	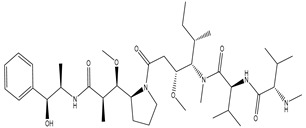	Metastatic cervical cancer	Microtubule-disrupting agent (in conjugation with antibody)	Approved in 2021

## Data Availability

Not applicable.
